# Understanding trial designs and acceptability of participation in an HIV vaccine trial with concurrent randomisation to oral pre-exposure prophylaxis in East and Southern Africa: a longitudinal qualitative study

**DOI:** 10.1186/s13063-026-09774-5

**Published:** 2026-05-16

**Authors:** Rachel Kawuma, Sarah Nakamanya, Edith Tarimo, Rujeko Samanthia Chimukuche, Jane Ambindwile, Doreen Pamba, Sylvia Kusemererwa, Patricia Munseri, Nishanta Singh, Karine Dubé, Sheena McCormack, Eugene Ruzagira, Janet Seeley, the PrEPVacc trial team, Rachel Kawuma, Sarah Nakamanya, Edith Tarimo, Doreen Pamba, Sylvia Kusemererwa, Patricia Munseri, Nishanta Singh, Sheena McCormack, Eugene Ruzagira, Janet Seeley, Glenda Gray, Zakir Gaffoor, Neetha Morar, Thandiwe Sithole, Kubashni Woeber, Samantha Siva, Eldinah Hwengwere, Rujeko Samanthia Chidawanyika, Nteboheleng Mahapa, Phindile Khanyile, Lucas Maganga, Wiston William, Emmanuel Kapesa, Elizabeth Danstan, Marco Missanga, Amani Kway, Abisai Kisinda, Lilian Njovu, Lwitiho Sudi, Revocatus Kunambi, Said Aboud, Eligius Lyamuya, Frank Msafiri, Agricola Joachim, Diana Faini, Tumaini Nagu, Deus Buma, Muhammad Bakari, Pontiano Kaleebu, Freddie Mukasa Kibengo, Ayoub Kakande, Jennifer Serwanga, Christian Hansen, Sheila Kansiime, Sylvia Masawi, Vincent Basajja, Shamim Nabukenya, Gertrude Mutonyi, Ilesh Jani, Edna Viegas, Isabel Remane, Odete Bule, Edna Nhacule, Patricia Ramgi, Raquel Chissumba, Eduardo Namalango, Yolanda Manganhe, Carmelia Massingue, Igor Capitine, Jorge Ribeiro, Jonathan Weber, Cherry Kingsley, Tom Miller, Angela Crook, David Dunn, Henry Bern, Aminata Sy, Simona Salomone, Liz Brodnicki, Sarah Joseph, Claire Wenden, Giuseppe Pantaleo, Song Ding, Charlotta Nilsson, Arne Kroidl, Julie Fox, Gustavo Doncel, Allison Matthews, Jim Rooney, Carter Lee, Merlin Robb, Kundai Chinyenze, Jacqueline Musau, Mabela Matsoso, Mary Amondi, Ansuya Naidoo, Paramesh Chetty, Anne Gumbe, Silindile Zulu, Londiwe Shandu, Tausi Sade, Gift Gadiel, Joel Ambikile Seme, Masunga Iseselo, Georgina Nabaggala, Esther Awino

**Affiliations:** 1 MRC/UVRI and LSHTM Uganda Research Unit, Entebbe, Uganda; 2 Muhimbili University of Health and Allied Sciences, Dar Es Salaam, Tanzania; 3 Africa Health Research Institute, Durban, KwaZulu-Natal South Africa; 4 Division of Infection & Immunity, University College London, London, UK; 5 National Institute for Medical Research-Mbeya Medical Research Centre, Mbeya, Tanzania; 6 Viral Pathogens and Epidemiology Interventions, MRC/UVRI and LSHTM Uganda Research Unit, Entebbe, Uganda; 7 HIV and Other Infectious Diseases Research Unit, South African Medical Research Council, Durban, South Africa; 8 Perelman School of Medicine, University of Pennsylvania, Philadelphia, PA 19104 USA; 9 MRC Clinical Trials Unit, University College London, London, UK; 10 Faculty of Epidemiology and Population Health, London School of Hygiene and Tropical Medicine, London, UK; 11 Department of Global Health and Development, London School of Tropical Hygiene and Medicine, London, UK

**Keywords:** Trial participation, HIV prevention, Knowledge, Acceptability, Implementation, Africa

## Abstract

**Introduction:**

This qualitative study was conducted to assess participants’ understanding of the PrEPVacc trial design and factors that influenced decisions to participate in the trial. PrEPVacc was a phase IIb, three-arm, two-stage HIV prophylactic vaccine trial with a second randomisation to oral pre-exposure prophylaxis. The main objective was to assess the safety and efficacy of two HIV-1 prophylactic vaccine regimens, each compared to placebo, in preventing the acquisition of HIV.

**Methods:**

Using a longitudinal approach, qualitative data were collected between October 2021 and September 2023. A sample of 105 participants (7% of trial participants) was purposively selected to participate in three in-depth interviews at 2, 6, and 12 months during the trial. Another 111 (92 females and 19 males) participated in 14 focus group discussions across four sites. Data were analyzed thematically.

**Results:**

Repeated information sharing sessions facilitated by health workers helped participants understand key trial concepts and the design. Pre-exposure prophylaxis (PrEP) was widely recognised as a protective measure alongside experimental vaccines. While vaccines were preferred for convenience and minimal side effects, individual perceived HIV risk was a main motivation for adhering to PrEP. Supportive relationships with health workers and close family members encouraged engagement in the trial, including acceptance to use the products. However, misinformation and stigma in communities created barriers to participation, with some participants facing social harm.

**Conclusion:**

Overall, participants demonstrated a good understanding of randomisation, double-blinding, and placebo control, facilitated by clear, repeated communication from health workers. Strengthening strategies to enhance informed consent, reduce stigma, and promote trial acceptance is crucial for successful implementation and retention.

**Clinical trial registration:**

ClinicalTrials.gov NCT04066881. Registered on December 15, 2020.

**Supplementary Information:**

The online version contains supplementary material available at 10.1186/s13063-026-09774-5.

## Background

Understanding clinical trial designs is important for informed consent [1], to ensure participants grasp the nature of research they are taking part in, including the randomization process, and potential risks [2, 3]. Together, these provisions contribute to trial acceptance, including uptake of investigational products.

**Fig. 1:**
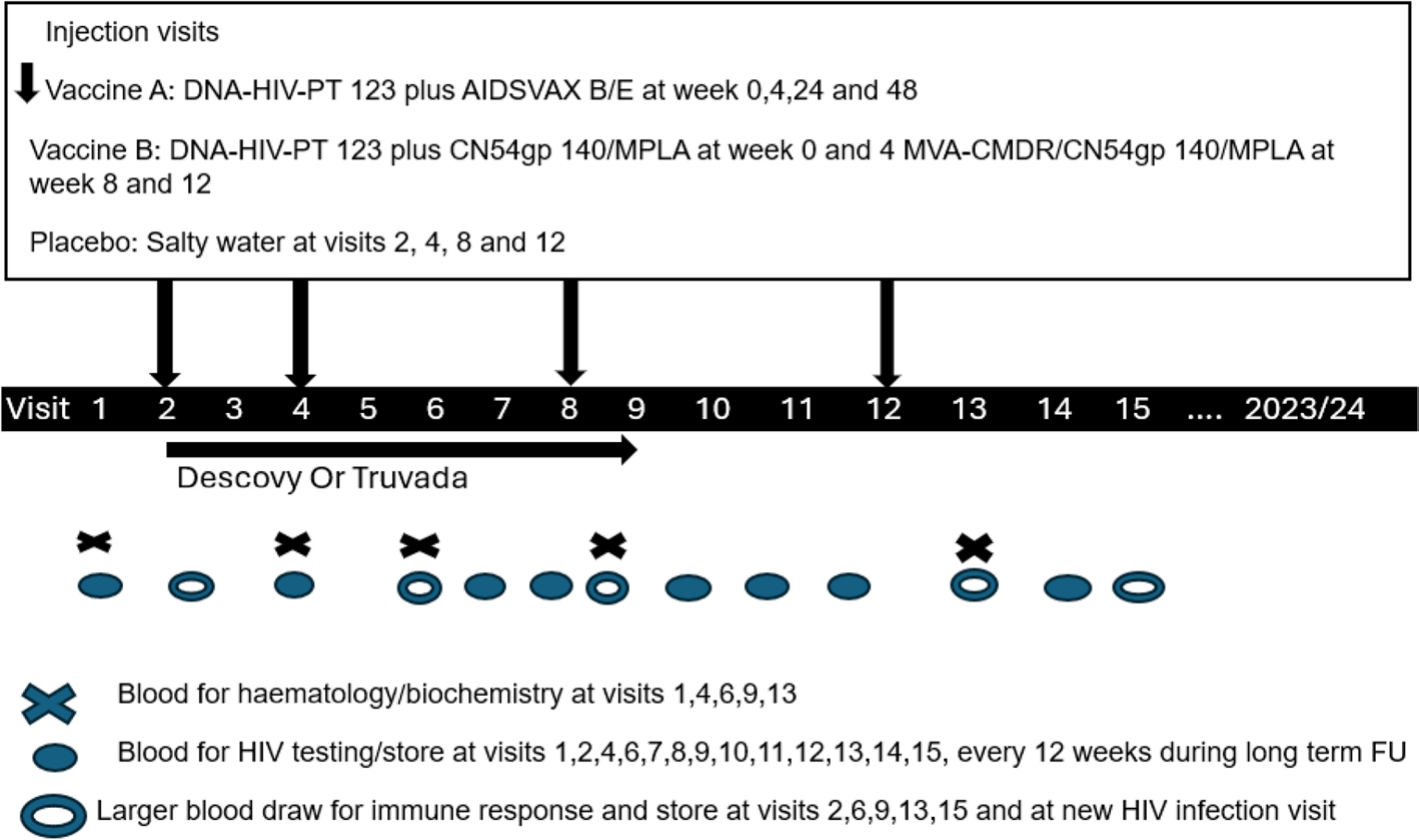
PrEPVacc trial schema

The PrEPVacc trial (Trial registration: NCT04066881) was a phase IIb three-arm, two-stage HIV prophylactic, vaccine trial with a second randomization to oral pre-exposure prophylaxis (PrEP). The main objective of the trial was to assess the safety and efficacy of two HIV-1 prophylactic vaccine regimens (DNA-AIDSVAX and DNA-MVA-CN54gp), each compared to placebo in a 1:1:1 ratio, in preventing the acquisition of HIV. PrEPVacc was a double-blind trial where participants had to adhere to vaccination for at least 3 time points to be included in the vaccine endpoint analysis. In addition, the second randomization compared the safety and effectiveness of a combination of tenofovir alafenamide/emtricitabine (F/TAF) relative to tenofovir disoproxil fumarate/emtricitabine (F/TDF) as oral PrEP in reducing HIV incidence, in the context of counterfactual background incidence (Fig. 1). Randomization to oral PrEP was open label, at a ratio of 1:1 but the allocation was only revealed at the point of first dispense to participants who agreed to take study PrEP. The PrEP was provided to them for approximately 6 months from randomization. Thereafter, participants were encouraged to obtain non-trial oral PrEP (F/TDF) from locally available sources. A qualitative study was embedded within the trial with the broader aim of exploring participants’ lived experiences and perceptions of taking part in an HIV vaccine trial with oral pre-exposure prophylaxis (PrEP) provided for prevention.

The inclusion of effective prevention methods like PrEP in HIV vaccine trials is an ethical obligation grounded in the principles of beneficence and non-maleficence [4], to ensure that participants without HIV are protected from HIV acquisition. However, this presents unique challenges, such as assessing the efficacy of the vaccine candidates in the presence of efficacious interventions [5] and ensuring that such a trial design is understood and accepted by the communities where it is being implemented [2, 6].

While qualitative research has investigated the acceptability of HIV prevention clinical trial participation and the factors that influence it [7–9], there has been a limited focus on evaluating participants’ understanding of trial designs, as a way to enhance clinical trial participation. In this paper, we examine how personal interpretations of the trial design, which had both experimental and effective products, influenced decisions to take part in the trial. Specifically, we explored: (1) how participants used local meanings to understand concepts of randomization, double-blind and placebo control, and why PrEP was included in the trial, (2) factors that supported understanding of the design and whether perceptions of the products provided in the trial influenced uptake, particularly the tension between using the experimental vaccines and PrEP provided at the same time, and (3) the role of external factors such as influences from partners, family members, and the community in making decisions regarding trial participation.

### Conceptual framework

**Fig. 2:**
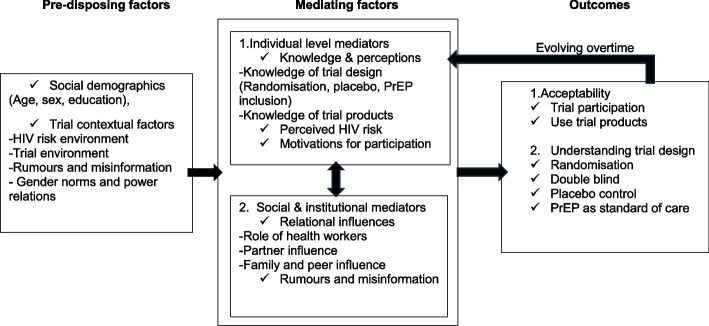
Conceptual framework mapping factors which influence trial understanding and acceptability (adapted from Tolley et al. 2018)

To guide our analysis, we developed an a priori conceptual framework (Fig. 2) adapted from the adherence framework proposed by Tolley and colleagues [10]. The original framework focused on adherence screening and monitoring in HIV prevention trials. We adapted the domain-based structure of that framework to examine how contextual and social factors such as participant relations, embedded within broader structural and relational contexts in the wider community and environment, interact to shape people’s attitudes, beliefs, and practices [11]. The framework is organized into three interconnected domains: predisposing factors, mediating factors, and outcomes. Predisposing factors include demographic characteristics, HIV risk context, and the broader social and environmental setting in which the trial takes place. Mediating factors comprise influences that directly support or hinder understanding of the trial design. These include knowledge about trial procedures which has been identified as crucial to understanding trial designs, and the decision to take part [3, 12], and individual perceptions of risk, known to influence acceptance of participation and use of products [8, 13]. Other factors that may influence clinical trial acceptability and the use of HIV prevention products include the role of health workers and influence from close relationships such as partners, peers, and family, as well as rumors and misinformation from the wider community in which the trial was taking place. Together, these contribute to the overall outcome of the present study which is participants’ understanding of the trial design and their agreement to participate, including their willingness to use trial products.

## Methodology

We used a longitudinal study design to assess factors that influenced trial acceptance, including decisions to take part, use of the experimental trial products, and understanding of trial design.

### Participant recruitment

Between December 2020 and March 2023, the trial team enrolled adults aged 18–40 years, at risk of acquiring HIV at four sites: in South Africa, Tanzania (two sites), and Uganda. In South Africa, the trial was conducted at the South African Medical Research Council site in Durban (site 1). Participants were recruited from the general population from areas with high HIV prevalence in the KwaZulu-Natal province. In Tanzania, the two sites were Muhimbili University of Health and Allied Sciences (MUHAS) in Dar es Salaam (site 2) and National Institute for Medical Research (NIMR)—Mbeya Medical Research Centre (MMRC) in Mbeya, southwestern Tanzania (site 3). Participants at MUHAS were bar workers and female sex workers, while the Mbeya site recruited female bar workers. The fourth site was in Uganda, based at the Medical Research Council/Uganda Virus Research Institute & London School of Hygiene and Tropical Medicine (MRC/UVRI & LSHTM) Uganda Research Unit in Masaka district, southwestern Uganda (site 4). Fisherfolk, female sex workers, and other groups at higher risk for HIV acquisition were recruited at this site.

HIV risk was assessed using a standardised questionnaire; participants were considered at higher risk of HIV acquisition if they reported any of the following during screening: suspected/confirmed sexually transmitted infection (STI), condomless sex with ≥2 partners or condomless sex with a new partner in the past 3 months, or condomless sex in exchange for money/goods in the past 3 months, and no prior use of PrEP.

Eligible participants were randomized to both the experimental HIV vaccines and oral PrEP and followed up as per scheduled protocol visits. Participants were encouraged to use condoms during sexual intercourse for STI and HIV prevention. Those of childbearing age were asked to adhere to contraceptive methods provided at the trial site or at a nearby health center from enrolment through to 18 weeks after the last injection, mainly because the effect of using an experimental vaccine on an unborn child was unknown.

### Sample size and data collection

We purposively selected a sample of trial participants at each site to take part in the qualitative study. Recruitment for the qualitative study occurred alongside that of the main trial. Participants were selected to provide a sample which included people with different characteristics such as age, sex at birth, highest level of education, occupation, PrEP arm (F/TAF versus F/TDF), and PrEP adherence behaviour according to self-report and urine test results, obtained from the main trial data.

Qualitative data collection using in-depth interviews (IDIs) and focus group discussions (FGDs) was conducted at all participating sites between October 2021 and September 2023. Each participant was to take part in three IDIs. The first was at month 2 of trial participation, aimed at collecting information on the participant’s life history, assessing perceptions of HIV risk and baseline knowledge of HIV prevention including experimental HIV vaccines and oral PrEP. The second IDI was scheduled at the end of the study PrEP period at month 6, and the third IDI was scheduled at month 12. At the second and third IDIs, we collected information on any changes in a participant’s HIV risk perception, knowledge, and experiences of trial participation, including use of experimental vaccines and oral PrEP. For the IDI group, gaps identified in the data collected were filled during subsequent interviews. In total, we recruited and interviewed 105 participants for the first IDIs across the four sites. During the subsequent rounds 2 and 3, some participants were lost to follow-up mainly due to relocation from the trial area and could not be contacted despite efforts to do so. Therefore, 94 and 90 out of 105 participants took part in IDIs at rounds 2 and 3 respectively.

We recruited other participants who did not take part in IDIs to participate in FGDs. These were divided into categories of participants who were adhering well to PrEP (good adherers) and those not adhering well (poor adherers); the sample was provided by trial statisticians from the main trial data base. The FGDs were conducted between weeks 30 and 48 during the trial. Altogether, 111 participants including 54 (45 women and 9 men) who adhered well to PrEP and 57 (47 women and 10 men) who did not adhere well to PrEP according to self-report and urine tenofovir results took part in 14 FGDs across the four sites.

In both IDIs and FGDs, participants’ understanding of the trial design was explored through open-ended questions asking them to describe what they understood about randomization, the use of multiple vaccine regimens, and the inclusion of oral PrEP as standard of care. Where relevant, interviewers probed participants’ understanding of the scientific rationale for comparing products and whether uncertainty about vaccine effectiveness had been explained to them. Participants were further asked whether their understanding of these design elements influenced their decision to enroll and continue participating. Data were collected by male and female interviewers with training in qualitative data collection and analysis techniques. A similar topic guide was piloted and used to collect data across the four trial sites. Prior to any data collection, information about the trial and the qualitative study, including products used and procedures involved, was shared with participants by the qualitative study team, followed by an opportunity to ask any questions for clarity before requesting informed consent to participate. Separate informed consent was sought from each participant to take part in each IDI and FGD they participated in. IDIs and FGDs were conducted in a private space at the research clinic, and each IDI lasted between 45–60 min while FGDs lasted between 60–90 min. IDIs and FGDs were conducted in a local language that was understood by the participants in each country and audio recorded. Regular debriefing meetings were held amongst interviewers at each site after every IDI and FGD to ensure completeness of the data.

Besides IDIs and FGDs, we conducted periodic structured debriefs with community engagement and counseling trial staff at each site, who were directly involved in recruiting and supporting participants to take part and use trial products. Structured debriefs typically refer to organised discussions or meetings that follow a format or set of guidelines [14]. The aim was to understand participants’ experiences and devise means to support them to continue taking part in the trial. The staff debriefs were moderated by social science interviewers. Anonymised feedback from the debriefs was shared with trial coordinators at each site and the trial management team to improve the conduct of the trial, including retention.

### Data management and analysis

All audio-recorded files were backed up on secure local servers at each site. Summary notes were written out shortly after conducting each IDI and FGD to ensure that a record was obtained. Thereafter, the full IDI or FGD was transcribed and translated into English. All scripts were anonymised with identification numbers to ensure confidentiality. A copy of the summary and final transcript was backed up on each site’s local servers with restricted access and on the main server hosted at LSHTM which housed the coordinating center. Notebooks were securely kept in lockable cabins and transcripts were stored on password-protected computers at the trial sites.

Data were analyzed manually, using a thematic framework analysis approach [15] covering three main topics, namely, knowledge about trial participation, factors that support understanding the design, and external influences of trial participation. A coding framework depicting the three topics above was developed jointly by all sites during monthly debrief meetings. Initially, site teams were asked to read two identical scripts and deductively code them by linking findings to the three topics. Emerging codes were discussed, revised, and agreed on to form a master coding framework developed in Microsoft Excel. Thereafter, each site team coded all their scripts by copying and pasting information and illustrative quotes against the matching topics or themes. For this paper, analysis was led by RK assisted by the lead social scientists at each site, SN, RSC, ET, JA, and DP, who organised data from the coding framework and identified relevant quotes to answer three main research questions related to (1) aspects of trial design that were understood, (2) factors that supported understanding of the trial design, and (3) external factors that influenced trial participation.

### Patient and public involvement statement

There was continuous interaction between the study team and the participants at different time points throughout the study. For instance, during participant meetings and clinic visits where clinic staff responded to any questions or concerns that participants raised. Besides the meetings, different services like HIV testing and counseling and family planning services were offered to the participants.

The study protocol was reviewed by a community advisory board (CAB) before submission to the regulatory bodies and implementation. The CAB also received periodic reports about the progress of the study and offered guidance on implementation and interpretation of results.

## Results

### Participants

Over 80% of study participants in both IDIs and FGDs were women; this was because two sites (2 and 3) recruited only female participants. Just over half (56%) of the participants were aged between 25 and 34 years, 37% aged 18—24 years, with the remainder aged over 35 years old. Regarding education, more than half had attained at least secondary level of education (54.3%). Half of the participants were randomised to each PrEP arm (Table 1).

**Table 1:** Socio-demographic characteristics of IDI and FGD participants from four sites who took part in the qualitative study of the PrEPVacc trial (October 2021–September 2023)

Category	IDI participants *n* = 105					FGD participants *n* = 111				
Country (site)	Site 1	Site 2	Site 3	Site 4	Total (%)	Site 1	Site 2	Site 3	Site 4	Total (%)
Age (years)										
18–24	11	3	12	13	39 (37.1)	15	11	13	9	48 (43.0)
25–34	17	11	18	13	59 (56.3)	17	17	10	17	55 (50.0)
35+	2	1	0	4	7 (6.6)	1	3	0	4	8 (7.0)
Sex										
Men	9	0	0	15	24 (22.9)	6	0	0	13	19 (22.9)
Women	21	15	30	15	81 (77.1)	21	31	23	17	92 (77.1)
Education										
Primary	0	5	17	22	44 (41.9)	6	12	8	16	42 (38.0)
Secondary	26	10	13	8	57 (54.3)	17	19	13	13	62 (56.0)
Tertiary+	4	0	0	0	4 (3.8)	4	0	2	1	7 (6.0)
PrEP allocation										
Descovy (TAF/FTC)	17	8	12	16	53 (50.5)	–	–	–	–	–
Truvada (TDF/FTC	13	7	18	14	52 (49.5)	–	–	–	–	–
PrEP adherence										
Good adherence	–	–	–	–	–	21	15	8	13	54 (50.5)
Poor adherence	–	–	–	–	–	6	16	15	17	57 (49.5)
Relationship type										
Single^*^	24	13	20	10	67 (64)	3	22	15	9	49 (64)
Married^**^	6	1	0	16	23 (22)	24	9	8	16	57 (21)
Divorced/separated	0	1	10	4	15 (14)	0	0	0	5	5 (15)
Occupation										
Formal work	5	0	20	2	27 (25.7)	2	0	0	2	4 (3.0)
Self-employed	5	0	6	20	31 (29.5)	5	0	5	12	22 (20.0)
Unemployed^***^	20	15	4	8	47 (44.8)	20	31	18	16	85 (77.0)

^*^Include those in relationships but may not be living together

^**^In a relationship and living together with partner as husband and wife

^***^Includes those who have no formal employment but earn income from informal arrangements such as sex work and casual labourers

In the following section, we present study findings from all four study sites guided by the conceptual framework (Fig. 2) to answer questions about which aspects of the trial design were understood and accepted, which factors supported or influenced this acceptance and the decision to participate.

### What aspects of the trial design were understood?

The longitudinal design of the study enabled the exploration of how participants’ understanding evolved over time. During baseline interviews, participants across the four sites described their limited comprehension of key elements of the trial design like randomization, double-blind, placebo control, and the rationale for including oral PrEP in a vaccine trial. Repeat information sessions provided by staff contributed to the clarification of these concepts as one participant from Tanzania reflected:

At first, I did not understand well about the study, but when I came for the second time, I got interested and understood well about the study and its importance. (Female, 23 years old, site 3, Tanzania)

Over time, participants could articulate these concepts more accurately, describing randomization as ‘everyone who was recruited in the trial having a similar chance to receive either an active or non-active (placebo) vaccine’. The randomization process was articulated by one participant as follows:

At the beginning of this trial, we were given training that there will be two groups; some will be given the real vaccine while others will receive the placebo, but you will not know if you will get the placebo or the real vaccine. (Female, 27 years old, site 2, Tanzania)

And double-blind was understood as ‘neither the staff nor participants knew which arm one was allocated to’ as illustrated below.

Even the people [health workers] who are giving us the injections don’t know [blinded] what drugs they are administering, whether it’s the real active drug/vaccine or a placebo. (Female, 22 years old, site 4, Uganda)

As a result, the rationale for adding PrEP as standard of care in the trial was understood as being to protect participants from acquiring HIV.

PrEP is given because there is no proof that the vaccine we are injected with can prevent HIV. It is still in research. (Female 29 years old, site 3, Tanzania)

Now we do take the vaccine together with the pills for maximum protection. (Female 23 years old, site 1, South Africa)

Overall, most participants understood that the vaccines were experimental and the trial included a placebo group, and that oral PrEP could protect all trial participants if used correctly and consistently.

### Acceptability of trial participation and product/s use

Besides understanding the design, we explored participant’s views about taking part in the trial and their decisions to use the products provided. We assessed how participants perceived the two products in the trial, vaccines (experimental product) and PrEP (standard of care) and whether their perception of one product (the vaccine) influenced acceptability to use the other (oral PrEP). We found that the decision to use either product was driven by experiential and contextual factors rather than knowledge alone. Although most participants consistently recognized that the vaccine’s efficacy was unproven and that PrEP offered protection if taken correctly, preference for the injectable vaccine persisted throughout follow-up due to perceived convenience, having fewer side effects, and being administered less frequently than daily oral PrEP.

To me the injection (vaccination) is much better. Even if it will be every day, that would be much better than these medicines (PrEP) which cause nausea, and you vomit until the ribs ache. (Female, 24 years old, site 2, Tanzania)

I just prefer the injection (vaccine) because I know that I will get it every after three months or after two months unlike the pills which you must take every day. (Female, 25 years old, site 1, South Africa)

In summary, acceptability of trial participation and product use was largely shaped by lived experience, convenience, and embodied responses to the products.

### What factors influenced understanding of the trial design and acceptance of vaccines and PrEP?

We explored both internal and external factors that facilitated or hindered understanding about the trial and use of the products. Internal factors included repeated sharing of information by trial staff and individual perceptions of HIV risk. External factors were the influence from people close to trial participants, rumours and misinformation circulating in the wider community, as explained in the following sections.

### Sharing information by trial staff

We found that repeated information sharing by the health workers supported comprehension of key trial elements and participation in the trial. This helped to equip participants with information and alleviated any fears or concerns they had, thus contributing to understanding of the trial design and supporting decisions regarding participation.

Every time I happen to have any question/fear/worries, for example after reading the informed consent document, I always come and ask the health worker, and I always find confidence in the reply to continue participating. (Male, 20 years old, site 4, Uganda)

The care I get from research staff, they are very quick to respond to our calls when we have something to ask or to report. (Female, 29 years old, site 3, Tanzania)

### Individual perception of HIV risk

In some cases, ongoing evaluation of personal HIV risk influenced continued PrEP use more than evolving knowledge about the scientific rationale of combined prevention. Risk was evaluated in relation to the number of sexual partners one had, sometimes of unknown HIV status, which increases the likelihood of acquiring STIs as explained below.

I don’t trust the women I sleep with and that is what encourages me to take PrEP. (Male, 32 years, site 4, Uganda)

Sometimes we don’t have the time to wear a condom, or you may not even know the [HIV] status of the person you are going to have sex with. (Female, 22 years, site 1, South Africa)

I am in multiple relationships, and my husband too, so the risk of getting HIV is high. (Female, 23 years, site 4, Uganda)

### Influence from close relationships

Decisions to take part in the trial were influenced by participants’ close relationships. Once they joined the trial, participants disclosed to significant people in their lives such as partners, peers, family members, and workmates. In many instances, they supported them to take part by reminding them about trial visits, facilitating them to attend visits and others simply gave words of affirmation, such as the support from partners:

Yes, he [partner] supports me and sometimes if he is not busy, he drives me here [research site] or I will tell him if I am through with study procedures and he will come to pick me up. (Female, 28 years, site 1, South Africa)

My partner is okay with my participation, and he allowed me to participate. When I joined the study, at first, I did not have a phone, and I gave his number to a secretary as my contact number. (Female, 21 years old, site 3, Tanzania)

However, in some instances, participants were discouraged from taking part in the trial by their partners resulting in conflict:

That is how the man [partner] started changing and separated with me and even changed his telephone numbers… he once called and said, ‘I loved you but lost interest when I heard about your participation in the study/trial’… That is how he ended the relationship, yet he is the man who used to give me enough money. (Female, 27 years old, site 4, Uganda)

I shared that I am participating in a study with the father of my child and the first thing he thought of was that I am HIV positive and had infected him. This resulted into a misunderstanding, and we did not talk for about two days until he went to test for HIV and realized that he was HIV negative. (Female FGD, site 3, Tanzania)

Besides participation, some partners dissuaded participants from using the trial products, particularly PrEP:

We used to quarrel that I am busy taking PrEP pills which means that I don’t trust her and so forth. She even insisted that I stop taking it so that we can trust each other. (Male 24 years, site 1, South Africa)

### Rumours and misinformation

From the wider community, there were rumors and misinformation about the trial and the products used which in some cases affected participants’ decisions to take part. For example, some rumors were due to misinformation about how the vaccines work and what the vaccines might do to a participant’s body:

People say that we will be infected with HIV viruses [we will acquire HIV] through the vaccine. Others say we are infected with the HIV virus [have HIV] that is why we are provided with the pills [PrEP]. (Female, 19 years, site 2, Tanzania)

Some people even said the research is aimed at extracting our body organs like the liver, the vaccines may damage our body systems, or we may fail to give birth. This scared me because I only had one child and if I failed to give birth, my husband would chase me. What scared me the most was the extraction of our liver. (Female, 26 years old, site 4, Uganda)

Participants who had just joined the trial and had not grasped the details of design were particularly susceptible to this misinformation, and initially this affected their decision to take part. However, with repeated counseling sessions, they were better equipped to counter community misconceptions and agreed to take part in the study:

At first, I was fearful because of the rumours in the community but after getting proper information from the clinic, all my fears went away and that is why I decided to participate in the trial. (Female, 24 years old, site 3, Tanzania)

Overall, our results show a multifaceted interplay of individual, interpersonal, and structural factors shaping trial participation and the acceptability of experimental HIV vaccines and oral PrEP, primarily driven by convenience and perceived biomedical efficacy across the four sites. While we noted a general understanding of the trial design, variations in the acceptability of products were noted among individuals and sites.

## Discussion

Our findings highlight that the understanding and acceptability of the trial were influenced not only by individual comprehension but by relational and structural contexts. The findings provide insights into how participants’ comprehension of the trial design, alongside influences from health workers and family members, informed decisions to join the study and to use both the experimental vaccine and oral PrEP (standard of care).

The longitudinal design of the study, with interviews at baseline, mid-point, and after 1 year, allowed us to examine how understanding evolved over time rather than treating knowledge as static. Across all four sites, participants demonstrated progressively stronger articulation of core trial concepts such as randomization, double-blind procedures, placebo control, and the rationale for including oral PrEP in the trial. While some participants initially reported limited understanding at enrolment, repeated engagement with health workers and ongoing counseling strengthened trial literacy over time. Comprehension of trial messages was reinforced through repeated sharing of information by the health workers, consistent with earlier studies [1, 16, 17]. Health providers were mentioned as a trusted source of information as has been noted elsewhere [18, 19], and structured communication systems in the trial supported consistent messaging across sites. Further still, the regular staff debriefing sessions facilitated by the social scientists strengthened responsiveness to emerging concerns. This supports evidence that informed understanding in clinical trials develops through iterative communication rather than a single consent encounter.

Although participants at the four sites over time demonstrated a good comprehension of the trial design, the interpretation of the design and their engagement with the trial were shaped by contextual and social realities underscoring that standardised messaging alone is insufficient. In South Africa, concerns centered on convenience and discretion; in Tanzania, anxieties focused on rumors, fertility, and potential bodily harm; and in Uganda, fears of disclosure and relationship instability were prominent. Overall, we did not observe marked gender differences in levels of comprehension. However, women more frequently described partner influence as affecting participation through relational authority, risk negotiation, stigma, and the need for product discretion. Multi-country HIV prevention trials therefore require locally tailored communication strategies that address context-specific concerns such as fertility anxieties, partner dynamics, and product convenience to strengthen informed consent, minimize social harm, and support retention.

Inclusion of oral PrEP within a vaccine efficacy trial introduced some uncertainty for participants. Although PrEP was recognized as protective, the vaccine was preferred due to convenience and fewer side effects [7, 20]. On the other hand, continued PrEP use was shaped more by lived experience and perceived HIV risk [21] than by scientific reasoning alone. Participants who considered themselves at higher risk were more likely to accept and adhere to oral PrEP, even when they preferred the vaccine. This conditional acceptance has implications for future trial designs and highlights the need for ongoing evaluation of participants’ experiences with trial products [22]. In an evolving prevention landscape that now includes long-acting injectable cabotegravir [23] and lenacapavir [24] that have been proven effective, future vaccine trials may face increased challenges in communicating scientific uncertainty, comparative effectiveness, and the rationale for evaluating multiple prevention products within a single study. Clear and adaptive communication strategies will be essential to support informed participation while preserving scientific validity.

Finally, disclosure to close family members facilitated acceptance of both trial participation and use of products [25–27], whereas misunderstanding and stigma sometimes generated conflict and, in extreme cases, social harms such as relationship breakdown as has been reported elsewhere [28]. Rumors and misinformation within communities further influenced participation, fostering mistrust and affecting retention [29, 30]. These findings highlight the importance of proactive community engagement, transparent communication, and gender-responsive counseling strategies that extend beyond individual-level information provision.

## Strengths and limitations

This study provides insights into how social context and gendered relationships influence understanding and acceptability of complex trial designs in multi-country biomedical trials. This was a qualitative methods study; the intention was depth rather than breadth by providing a contextualized understanding. Therefore, the study included a subset of the participants who took part in the PrEPVacc trial; consequently, the findings are not generalizable. The main strength of the study lies in the longitudinal data collection approach across four diverse research sites, which enabled exploration of how participants’ knowledge and experiences evolved over time and influenced understanding of the trial design and decisions to participate. In addition, sampling across multiple sites and participant groups allowed inclusion of different perspectives. While we acknowledge that the study was not designed to formally compare sites, the consistency of findings across all four sites suggests that key aspects of the trial design were well understood in the different trial settings.

## Conclusion

Our main finding was that repeated provision of clear and standardized information supported understanding of the trial design. However, while participants demonstrated a good grasp of key trial concepts, their preferences for the experimental vaccines over daily oral PrEP, known to be efficacious, highlight ongoing challenges in HIV prevention research. Future trials will need to adapt to an evolving prevention landscape, ensuring that participants have access to the most effective prevention methods while maintaining scientific integrity. Besides having knowledge about the trial design, participants’ decisions to take part were influenced not only by their perceived benefits but also social influences, and external barriers such as misinformation and social stigma.

To support successful trial implementation and product uptake, it is crucial to develop culturally appropriate and locally relevant communication about trial procedures, including risks and benefits. Ongoing education for participants, their close family and friends, and the broader community is essential to address rumors, build trust, and combat misconceptions. These efforts can mitigate barriers such as stigma and social harm, enhance the trial experience, and improve the credibility and acceptance of future HIV prevention trials.

## Supplementary Information

Supplementary Material 1.

Supplementary Material 2.

## Data Availability

Data are available upon reasonable request.
