# Supplementary material for: Understanding trial designs and acceptability of participation in an HIV vaccine trial with concurrent randomisation to oral pre-exposure prophylaxis in East and Southern Africa: a longitudinal qualitative study
Source: Trials. 2026 May 16;27:412. doi: 10.1186/s13063-026-09774-5 (PMC13231623; doi:10.1186/s13063-026-09774-5)
Supplement: Supplementary file 1 — Supplementary Material 1. [file 13063_2026_9774_MOESM1_ESM.docx]

**PrEPVacc main trial In-depth Interview (IDI)/Focus group (FGD) topic guide** **FOR TRIAL PARTICIPANTS version 1.0**

**Interviewer**: Start by explaining the following to participants.

- The IDI will be a 45mins-1 hr activity that will be audio-recorded.
- The FGD will last between 1-1and half hour and will be audio-recorded.
- We would like to hear your views. There are no right or wrong answers.
- Please feel free to give your ideas
- Any question you feel uncomfortable about, please feel free not to answer it. However, for purposes of learning, we would like to hear everything you want to say. Therefore, if this is of no harm to you, try and tell us everything.
- Your names will be kept confidential – when we write up the discussion, we never use people’s real names. Instead, we will allocate a number which we shall use to identify you.
- The information you share with me will be between the two of us (emphasise that even the clinic team will not know what participant tells you)
- I/we will be happy to answer any questions that may come up
- Please put your cell phone/s on silent if possible
- Is it okay with you that we start the discussion?

**NOTE: RECORD THE DATE AND TIME ACTIVITY STARTS**

**[For IDI]**

**Background Information**

[Age, Religion, Education, Marital status, job/Employment]

**Recent life history**

1. Please tell me a bit, about yourself?

- Places lived
- Relationship (family, married/single/any children and partners, present and past)

**[For FGD]**

Before the FGD starts, record some salient information from each participant such as [Age, Religion, Education, Marital status, job/Employment]

**Topic 1: Knowledge and participation in the study/ trial**

1. Tell me about the study you are participating in now?

*(Get participants to discuss what product they are using, why and how)*

- *Do they know about the randomisation in the study and what that means?*
- *What do you understand about how the study works?*
- *(Probe for understanding of vaccine, placebo, PrEP, follow-up procedures.)*
- *Do they know about the PrEP and Vac components in the study?*
- *How do you understand the reason for using PrEP in a vaccine trial?*
- *Why do you think participants are encouraged to continue taking PrEP even after receiving the vaccine?*
- *How do you personally balance the idea of taking a daily pill (PrEP) and receiving a vaccine that might protect you in the future?*

2. Why did you choose to participate in the study?

*(Probe about motivation, peer influence, past research experience, HIV/AIDS/STIs)*

**Topic 2: Knowledge/ Use and acceptability of PrEP**

1. What do you know about PrEP?

(ARVs, taken by negatives, prevention method)
2. What do you think about the pill that you are taking in this study?

*(Probe for appearance, taste, size, likes and dislikes, fears, are they able to tell whether they are on TAF or TDF)*

*3.* What do you think about using ARVs yet you are HIV negative (feelings; regrets, issues of stigma, side effects, etc)

1. People often miss pills they’re supposed to take on a regular basis. Are there times you have failed to take the pill as instructed? (probe around adherence)
2. What has made it easy/difficult to take the pills?*(barriers & facilitators)*
3. Does the pill protect against HIV/AIDS/STIs? *Reasons? (Probe on why s/he thinks about taking the pill yet they were vaccinated and are encouraged to use condoms)*

**Topic 3: Other people’s reactions (partners/ people around them (family, workmates, peers, etc)**

1. Is there anyone else who knows that you are participating in this study? Who?
2. What do your partners/ people around you (family, workmates, peers, etc) think about your enrolment in this study? (*Ask about positive things that happened, challenges, concerns, and fears from partner*/ people around them (family, workmates, peers, etc)
3. How has your partner/ people around you (family, workmates, peers, etc) supported your participation in the trial?

**Topic 4:** **Risk Perception *[Ask only IDI participants]***

1. **To be asked at first interview in the study:** Do you feel at risk of acquiring HIV/AIDS? Explain your answer.
2. **To be asked at follow up visits:** How is your perception of risk now, compared to when you started the study?

**Topic 5: Acceptability of study procedures**

1. What are your experiences with the diary used during the study? *(Easy, difficult, any other comments)*
2. What were your thoughts about the other study procedures (taking off samples, vaccination and examinations)?
3. Ask about pregnancy; why do you think you/your partner are encouraged not to become pregnant while participating in this study?

- How do you feel about this study requirement (probes for feelings of resentment from partner, discussions to postpone pregnancy and other issues)

1. Now, let us talk about your participation in the study

- What are the factors for your continued participation in the study?
- What could be that ‘thing’ that may cause you to drop out of the study?

**Interviewer:** End by encouraging the participants to ask any questions they may have; answer those you are able to and refer them for clarification of those you are unable to answer.

Now, thank the participants/s and close the discussion
